# The association between work-related factors and use of psychotherapeutic methods with burnout rates in family physicians

**DOI:** 10.1192/j.eurpsy.2021.1319

**Published:** 2021-08-13

**Authors:** L. Rubene, A. Miksons

**Affiliations:** 1 Psychiatry And Narcology, Riga Stradins University, Riga, Latvia; 2 Psychiatry, Riga Centre of Psychiatry and Narcology, Riga, Latvia; 3 Psychosomatic Medicine And Psychotherapy, Riga Stradins University, Riga, Latvia

**Keywords:** family medicine, burnout, psychotherapeutic methods

## Abstract

**Introduction:**

A physicians work is closely related to patients and the understanding of their problems. The use of psychotherapeutic methods is a factor for successful care (Swanson, 1994). At the same time burnout is a syndrome that can affect the health of doctors themselves thus reflecting on the quality of care they can provide (Lloyd et al, 2002).

**Objectives:**

To investigate the use of psychotherapeutic methods in family physicians work in Latvia and the association between burnout rates and the use of these methods in practice as well as different demographic and work-related factors.

**Methods:**

A cross sectional study was carried out. An anonymous online form that contained questions about the demographic data, psychotherapeutic methods/techniques mastering and usage and questions from the Maslach Burnout Inventory was sent out to the publicly available email addresses of family physicians in Latvia. The collected data was analyzed using Microsoft Excel and IBM SPSS software.

**Results:**

Together 54 responses were received from all regions of Latvia. The analysis found association between work hours per week, patients seen per week, usage of psychotherapeutic methods and burnout. More hours per week was associated with higher rates of depersonalization (p=0,014) and burnout (p=0,010). More patients per week was associated with higher rates of burnout (p=0,024). Being unsure if they were using any psychotherapeutic methods was associated with higher rates of depersonalization (p=0,028).

**Conclusions:**

The data obtained allows a better insight in to the usage of psychotherapeutic methods, everyday work and the association with burnout rates in family physicians.

